# Responses of adult spotted lanternflies to artificial aggregations composed of all males or females

**DOI:** 10.3389/finsc.2022.981832

**Published:** 2022-09-08

**Authors:** Miriam F. Cooperband, Kelly Murman

**Affiliations:** Forest Pest Methods Laboratory, USDA APHIS PPQ S&T, Buzzards Bay, MA, United States

**Keywords:** aggregation, sex ratio, attraction, trapping, pheromones, phenology, reproductive biology

## Abstract

Spotted lanternflies (SLF) *Lycorma delicatula* are economically important invasive planthoppers discovered in North America in 2014. SLF are gregarious, but how they locate each other, or who finds whom and when, is poorly understood. Here we describe adult SLF behavior and phenology on their preferred host, *Ailanthus altissima*, under field conditions, in the context of both aggregation and mate-location, since SLF demonstrated aggregation prior to mating. We documented aggregation behavior of adults and found we could manipulate free-living SLF populations in both number and sex ratio by the placement of confined populations of SLF males or females on trees. Trap capture of arriving SLF was significantly higher on trees with confined SLF aggregations than on control trees, and was corroborated with photographic data, demonstrating the manipulation of attraction and aggregation behavior. Sex ratios of trapped SLF arrivals were significantly more male-biased on trees with confined males and more female-biased on trees with confined females, evidence that the male- and female-biased sex ratios observed on trees naturally can be explained by sex-specific conspecific signals. SLF sex ratios shifted over time in the same pattern over two consecutive years. A mark-release-recapture study over time found that 1) SLF behavior is density dependent and strongly influenced by natural populations, 2) released females were captured significantly more on trees with caged females, particularly prior to mating, and 3) released males were captured significantly more on trees with caged females starting at mating time. Photographic data revealed that most clustering behavior (a measure of courtship) of free-living SLF began on trees with caged females during mating time, but not on trees with caged males or controls. We describe adult male and female SLF phenology whereby 1) aggregation behavior occurs, 2) males and females arrive at different times, 3) females began to aggregate several weeks prior to mating, 4) males subsequently joined aggregations at the time of mating, and 5) aggregation continued into oviposition. Population density and aggregation behavior were found to be key factors in their natural history which can be manipulated, providing a foothold for future research. Possible mechanisms for future exploration are discussed.

## Introduction

Spotted lanternfly, *Lycorma delicatula* (White) (Hemiptera: Fulgoridae) (hereafter, SLF), is a phloem-feeding invasive pest, with a broad host range, that has spread to numerous U.S. states since its first detection in eastern Pennsylvania in 2014 (1–3). With populations expanding relatively unchecked, they occur in large numbers, and their intensive feeding causes direct damage to and even death of host plants, particularly grapevines, posing a significant threat to the grape industry (4, 5). Indirect damage occurs when heavy SLF feeding in trees causes honeydew to rain down from the canopy, coating the understory, and promoting the growth of sooty mold which blocks photosynthesis, killing understory plants. Large SLF populations in urban and suburban areas, and the accumulation of their honeydew on patios, cars, and other outdoor items, in turn attracting stinging insects, impact outdoor activities and create a nuisance to humans. Around the time of mating, swarms of adult SLF take flight and have entered aircraft, manufacturing and packing factories, and food-processing facilities, and in some cases have rendered products unusable, causing problems for businesses (KM pers. obs., G. Parra, pers. comm.). Furthermore, cryptic SLF egg masses are deposited on outdoor objects, including timber, plant nursery stock, toys, furniture, tiles, rocks, vehicle wheel wells, shipping containers, and train cars, making them excellent hitchhikers and facilitating their spread to new areas (5). Thus, SLF threatens numerous industries, worth billions of dollars, through direct and indirect feeding damage, disruption of commercial activities due to their presence in large numbers, and quarantines restricting movement of infested goods. Until its invasion in the U.S., little information was available on SLF biology, and even less on its reproductive biology. In the last 8 years, researchers have begun to fill the knowledge gaps and develop tools to control this pest outbreak.

Although SLF are polyphagous, they have a strong association with tree-of-heaven *Ailanthus altissima* Swingle (Mill.) Swingle (Sapindales: Simaroubaceae) (3, 4, 6). In Pennsylvania, adults oviposit between the end of September and early November when they die, eggs overwinter, and nymphs start to emerge in the end of May or early June (3, 7). Each of the four nymphal stages lasts approximately two weeks, and the first adults emerge in the end of July. Nymphs are highly active, mobile, gregarious, and polyphagous, but as they develop, their diet becomes more specialized on their preferred host *A. altissima* (3, 6, 8). Adults are long-lived, and in the first six weeks prior to the observation of mating, described as “Early”, they predominantly can be found feeding (7, 9). About halfway into Early, near the end of August, large aggregations start to appear on *A. altissima* with honeydew accumulating and, at the bases of the most heavily-infested trees, becoming white and frothy, and emitting a strong smell of fermentation (2, 10). It is at this time when large numbers have also been observed to take flight (9, 11) and sex ratios have been observed to become strongly skewed, with mostly males on some trees and mostly females on other trees (12, 13). Mating is first observed in mid-September, marking the beginning of a stage called “Mid”, and a week or two later the first egg masses start to appear, marking the beginning of a stage called “Late” (7).

Tools for early detection typically combine powerful attractants, such as pheromones or kairomones, with effective traps (14). Numerous kairomones were recently identified for SLF (15), but no pheromones have been identified for SLF or any planthopper (5), although this may be due to lack of investigation. Bioassay studies produced evidence of possible pheromone use in SLF (MFC, unpublished) (16). Evidence to suggest that SLF may actively aggregate has also been found recently (17, 18). If aggregation or mating behavior in SLF is mediated by a pheromone, it could lead to the discovery of powerful attractants. Thus, our research efforts aim to determine: 1) where, when, and how adult SLF find each other, 2) if adult aggregation is actively taking place, 3) which sex releases signals and which sex responds to them, and 4) the timing and physiological state required by SLF to release these signals so that we can collect, study, and exploit them.

We sought to answer the question “Who finds whom and when?” under field conditions. Thus, in 2020, we conducted an experiment in the field using artificial aggregations of either male or female SLF adults confined in sleeve cages on trees, with circle trunk traps placed above them to capture the naturally occurring SLF responding to the confined populations. This experiment was designed to measure the number of naturally occurring adult SLF males and females arriving in response to aggregations of each sex, as well as marked-released-recaptured SLF with an equal opportunity to reach a tree with an artificially confined male or female aggregation. Based on resulting observations in which the trees with the artificial aggregations on them appeared to have triggered aggregation behavior of free-living SLF, the experiment was repeated in 2021 with the addition of control trees that had empty sleeves, and the collection of photographic data.

## Materials and methods

### Sleeves and traps

**Figure 1:**
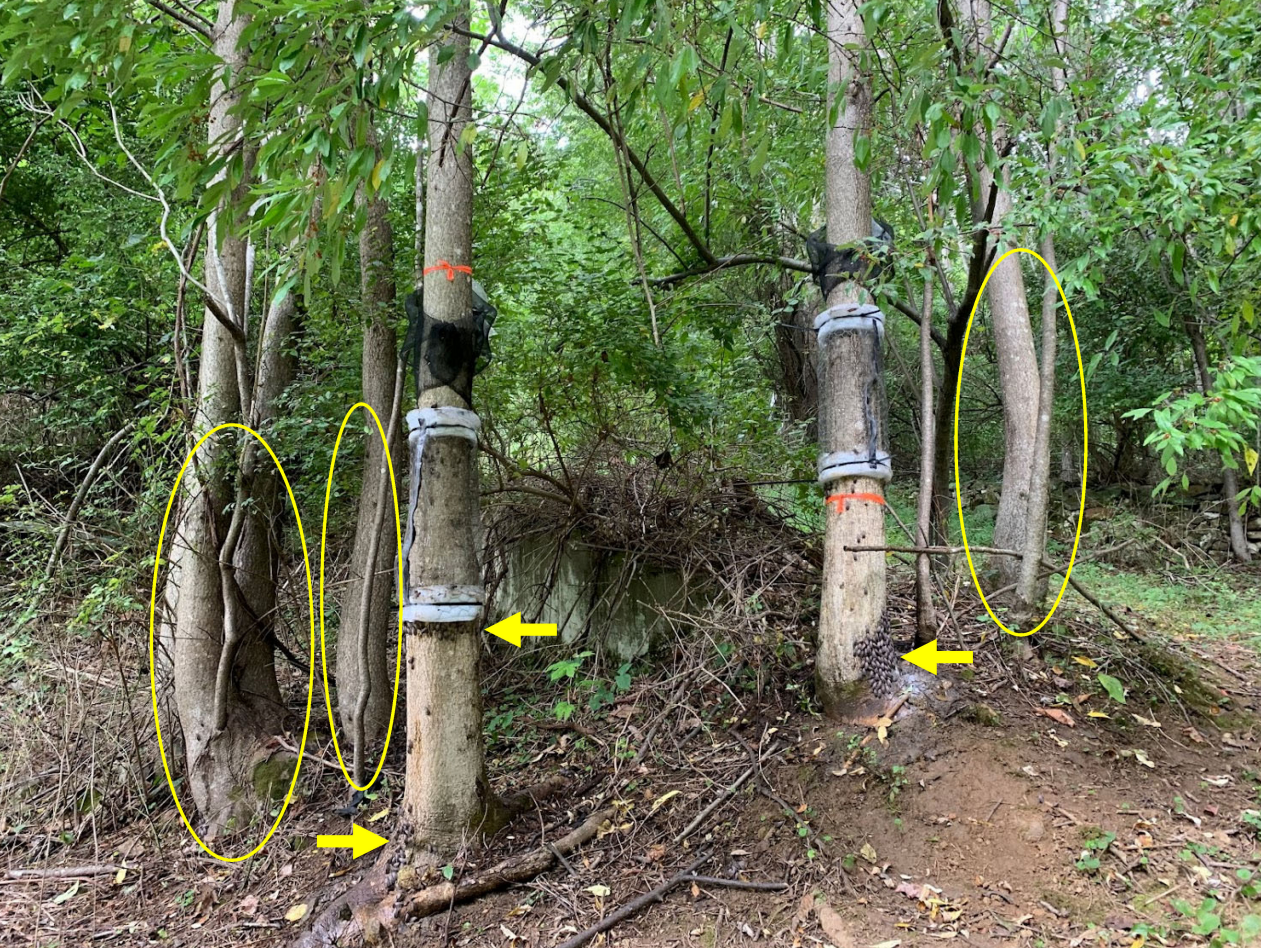
A photograph from 2020 showing two *Ailanthus altissima* trees with sleeves containing adult spotted lanternflies, *Lycorma delicatula* (SLF). One sleeve contained males and the other contained females. Natural aggregations of free-living adult SLF accumulated beneath both sleeves (arrows). The nearby *A. altissima* trees of similar size (circled) had no SLF aggregations.

Experiments, detailed in sections below, were performed in the field in 2020 and 2021 with blocks of either two or three trees, respectively. Sleeves containing either males or females were placed around tree trunks, and in 2021 there were also control trees with no SLF inside the sleeves. A circle trunk trap was placed above each sleeve (Great Lakes IPM, Vestaburg, MI) (19) with the bottom edge placed at breast height. Traps collected arriving SLF into a bag rather than a jar, which was found to be significantly more efficient at capturing SLF (20). A pesticide strip was placed in each trap bag and refreshed every six weeks to prevent escapes and predation (Vapona II 2,2-dichlorovinyl dimethyl phosphate (10%), Hercon Environmental, Emingsville, PA) (19). Two field experiments in consecutive years (2020 and 2021) tested the cumulative effects over time of placing artificial aggregations of males or females on paired trees in low density field sites. In both experiments, the artificial aggregations were confined within custom sleeves (76 cm tall) enclosed around trunks of *A. altissima* trees. The top of each sleeve started 2-3 cm below the bottom of the circle trap which captured free-living SLF that arrived on the tree trunk. Sleeves were constructed by first placing three layers of foam batting (BugBarrier; Environmetrics Systems USA, Inc., Victor, NY) around the trunk at the top and bottom margins of the sleeve to provide space between the sleeve and the trunk for the SLF inside to move around. Chicken wire was placed over the batting, followed by tulle mesh over the chicken wire. These were all secured to the tree at the top and bottom using zip ties, and the vertical seam in the tulle was closed using Velcro in 2020 (Velcro Companies, Inc., Manchester, NH), and yellow lab tape in 2021 (Research Products International, Mt. Prospect, IL) (**Figure 1**). At the beginning of the first week, sleeves were stocked with groups of live field-collected males or females (numbers and details described for each year below). At the beginning of each subsequent week, sleeve contents were checked, and if some died or escaped, they were replaced with newly captured SLF of the designated sex. Sleeves on the control trees in the 2021 blocks contained no SLF. If a sleeve or trap was found damaged, the whole block was excluded from analysis for that week. Weekly trapping was conducted from August 10 to October 26 in 2020, and from August 17 to November 3 in 2021, for a total of 11 weeks of trapping each year with the start date staggered by one week (**Table 1**).

**Table 1:** A description of the timing of spotted lanternfly, *Lycorma delicatula*, activities in the field, and numbers of trapping block replicates per week per year.

			Trapping blocks (N)		
**Stage**	**Week**	**Date Range**	**2020**	**2021**	**Primary activities observed on *A. altissima***
Early-1	1	8/10 – 8/17	10		Adults recently emerged, feeding
	2	8/17 – 8/24	10	10	Feeding continues
Early-2	3	8/24 – 8/31	9	10	Feeding continues, aggregations form, sex ratio sharply changes to female-biased
	4	8/31 – 9/7	10	9	Flight behavior increases
Mid	5	9/7 – 9/14	10	9	Sex ratio shifts back again, first observation of courtship and mating in the field
	6	9/14 – 9/21	10	10	Courtship and mating increases, first observation of oviposition in the field
Late-1	7	9/21 – 9/28	10	9	Oviposition increases, courtship and mating continue
	8	9/28 – 10/5	10	9	Oviposition increases and courtship and mating decreases
Late-2	9	10/5 – 10/12	10	10	Oviposition continues and courtship and mating decreases
	10	10/12 – 10/19	10	10	Oviposition continues and courtship and mating taper off
Late-3	11	10/19 – 10/26	10	10	Oviposition becomes most observed behavior
	12	10/26 – 11/2		10	Oviposition continues, death with freezing temperatures

Events denoting key physiological shifts, such as the first observations of mating and freshly oviposited egg masses, occurred approximately 5 calendar days later in 2021 than in 2020. Consequently, stage designations are slightly offset in the two years, but for purposes of labeling we use the stage designations from 2020.

### Capture of naturally occurring SLF on trees with artificial aggregations - experimental design in 2020

Rural field sites were located on private properties with forest edges in Warren County, NJ, selected for their pairs of similarly-sized and spaced *A. altissima* trees, as well as the presence of low density populations of SLF. This was determined in the early spring by visual inspection of each site by two experienced scouts, and finding only 1 egg mass or 1-10 nymphs in 15 min of searching. Seven sites in Warren County, NJ were selected to establish 10 blocks, each containing a pair of *A. altissima* trees spaced 2 to 3 m apart. In 2020, the average difference in diameter at breast height (DBH) between male- and female-sleeved trees in each pair was 4.2 cm, with the male-sleeved tree being the larger tree in 5 blocks, and the smaller tree in the other 5 blocks. The average tree DBH ( ± SE) was 18.6 ( ± 1.6) cm. Data from one block was discarded in week 3 due to weather knocking down a trap (**Table 1**). In 2020, each block consisted of two sleeved trees, one containing 40 adult male SLF and one containing 40 adult female SLF to answer the question “who finds whom and when?” based on the number of naturally occurring adult male and female SLF captured each week on male- or female-sleeved trees.

### Capture of naturally occurring SLF on trees with artificial aggregations - experimental design in 2021

A second experiment, conducted in 2021, attempted to duplicate the first experiment, but with the addition of a third *A. altissima* tree to each block, outfitted with a trap and an empty sleeve which served as a control to demonstrate what a normal wild, or naturally occurring, population would look like. The purpose of adding the control trees was to assess whether the presence of artificial aggregations resulted in wild aggregations. Field sites in 2021 consisted of a mix of private properties and state wildlife management areas, with forest edges. In 2021, 11 blocks were initially established on six rural properties; nine were in Sussex County, NJ and two were in Warren County, NJ. During the study, two blocks in Warren County, were abandoned due to bear activity. However, two additional blocks were established mid-study in Sussex County. Since other blocks already had established sleeves, the sleeves in the newly added blocks were allowed to establish for one week prior to data collection, resulting in a total of 9 or 10 blocks each week (**Table 1**). As in 2020, paired male and female trees in 2021 were 2 to 3 m apart except for one pair that was 3.5 m apart. The control tree represented either the third point on a triangle with the other two trees, or the third in a line if a suitable tree in the triangle position could not be found. Sites in 2021 were selected not only for their presence of triplets of similarly-sized and spaced *A. altissima*, but also the presence of low density populations of SLF. Prior to the experiment in 2021, populations were sampled with circle traps set on June 30, 2021, and captures of 30-40 SLF per site over a 5-week period indicated a low initial population density. In 2021, the average difference in DBH between male- and female-sleeved trees within each block was 1.6 cm, with the male sleeve being on the larger tree in half of the blocks and on the smaller tree in the other half of the blocks. Control trees were on average 4.1 cm DBH larger than the other trees in their blocks. The average DBH ( ± SE) of all trees used in 2021 was 19.7 ( ± 0.8) cm. The numbers of egg masses deposited inside the sleeves was noted weekly.

### Photographic data on SLF clusters

To record SLF that may have landed on trees without entering traps (see **Figure 1**), in 2021 a photograph of each tree was taken weekly from August 10 when sleeves and traps were first set up until October 27. Each photograph encompassed the tree trunk from the ground to just above the trap on any side where any SLF were seen. For each tree photograph, the total numbers of free-living SLF, and the numbers of clusters of free-living SLF, defined by two or more SLF physically touching each other, were quantified.

### Marked-released-recaptured SLF adults

In addition to investigating movements of naturally occurring SLF with respect to the artificial aggregations at low density sites, in both years a second study was superimposed at the same time and place, in which a known number of marked male and female SLF were released on the ground, halfway between the male-sleeved and female-sleeved trees, and their responses were recorded given their known starting point and an equal probability of arriving at either tree. Equal numbers of males and females were released each week, but in 2020, weekly releases varied between 10-25 of each sex (average of 16.4) released per block. In 2021, 15 SLF of each sex were released weekly between each male- and female-sleeved tree pair. Since the density of SLF naturally occurring on trees was an uncontrollable factor with the potential to influence where marked SLF arrived, and SLF density was found to contribute to SLF orientation in the field (MFC, unpublished) (21), the relative SLF density between the trees in each pair was taken into consideration in the final analysis. Each week, the number of naturally occurring SLF per cm circumference caught on each tree was counted and categorized into one of eight categories (<0.1, 0.1-0.5, 0.5-1, 1-2, 2-3, 3-6, 6-9, and 9-12 SLF per cm circumference of the tree at breast height). For each week, if one tree fell into a different density category than the other tree in its pair, they were considered to have different densities: higher and lower. If they were in the same density category, the trees in the pair were considered to have the same density. For each release, the combination of these density categories with the male sleeve vs. the female sleeve choice, were considered in the analysis of which tree in each pair the released SLF chose. Therefore, the following density-sleeve treatments were compared: higher-female vs lower-male, lower-female vs higher-male, or same-female vs same-male.

### Insects

At the beginning of each week, adult SLF were collected and sexed, sleeves were restocked, trap bags were changed, and SLF were marked and released. SLF were collected from *A. altissima* growing nearby (<30 km) private properties that were heavily infested with SLF and were free from pesticides. This ensured sleeves had the correct number of live SLF in them at the beginning of each trapping period. For the mark-release-recapture experiment, equal numbers of male and female SLF were dusted with fluorescent powder dye (DayGlo Color Corp., Cleveland, OH) and released on the ground halfway between the trees with male and female sleeves. A different color dye was used each week to determine how long ago the recaptured SLF had been released.

### Data analysis

The total naturally occurring SLF captured and their sex ratio (percent male), for the entire season on the paired male- and female-sleeved trees in 2020 were examined using a matched paired T-test (α = 0.05). Sex ratio data in 2020 were normally distributed, but season totals of males, females, and total SLF were not. Therefore, log transformation was used to normalize the data for the analysis of season totals. In 2021, with the addition of a third treatment to each block, totals for the entire season were log transformed, and sex ratios were arcsin-square-root-transformed, which normalized the data, which was then analyzed using ANOVA and Tukey means separation (α = 0.05). Back-transformed data are reported.

Weekly catch of males, females, and sex ratio was examined to expose patterns or changes over time. For this, a Wilcoxon test was used because data were not normally distributed due to many zeroes (α = 0.05). In 2021, for weeks showing significance, a Wilcoxon test was conducted on each pair with Bonferonni correction (α = 0.025).

Photographic data in 2021 were also not normally distributed. Data were consolidated into three periods based on the dominant behavioral activity, feeding (weeks 1-5), mating (weeks 6-9), or oviposition (weeks 10-12), and the number of clusters were compared by these time intervals, and by sleeve treatments, using Wilcoxon test and Bonferroni corrections (α=0.025). If found to be significant, Wilcoxon pairwise comparisons were performed, also with Bonferroni corrections (α=0.0125). The same analysis was conducted for total number of SLF per tree. All above analyses were conducted using JMP (v. 10.0.0).

For the mark-release-recapture study, due to low numbers of recaptured SLF, data for 2020 and 2021 were combined and grouped into three 4-week time periods as follows. Early-1 and Early-2 corresponded to the first four weeks of data collection from August 10 to September 7 when feeding was the primary activity and mating had not yet been observed in the field. Mid and Late-1 corresponded to the second four weeks of data collection from September 7 to October 5 when mating was observed in the field and was the primary activity, but it included the beginning of oviposition. Finally, Late-2 and Late-3 corresponded to the final four weeks of data collection from October 5 to November 2, when oviposition was the primary activity in the field, courtship and mating activity tapered off, and adults began to die (**Table 1**). The *post hoc* analysis categorized the treatments into groupings based on whether one tree in a pair had higher, lower, or the same naturally occurring SLF background density relative to the other tree in the pair that week, as described above. Because each insect released equidistant between two trees had an equal chance of arriving at either tree, a chi-square test was used to test the null hypothesis that released male and female SLF would arrive at the male-sleeved and female-sleeved trees with equal frequency (α = 0.05 with G ≥ 3.84) (22).

## Results

### Phenology

A general phenology of observed activities is described in **Table 1** with definitions of the adult phases, names given to each two-week period, and the number of replicates acquired in each week and year. Developmental stages in 2021 lagged behind those in 2020 by approximately 5 calendar days.

Mating in the field was first observed on September 8 and 13, in 2020 and 2021, respectively, marking the onset of the “Mid” stage. Approximately one week later, on September 16 and 20, in 2020 and 2021, respectively, the first freshly oviposited egg masses were observed in the field, and mating activities began to overlap with oviposition activities.

### Capture of naturally occurring SLF on trees with artificial aggregations

**Figure 2:**
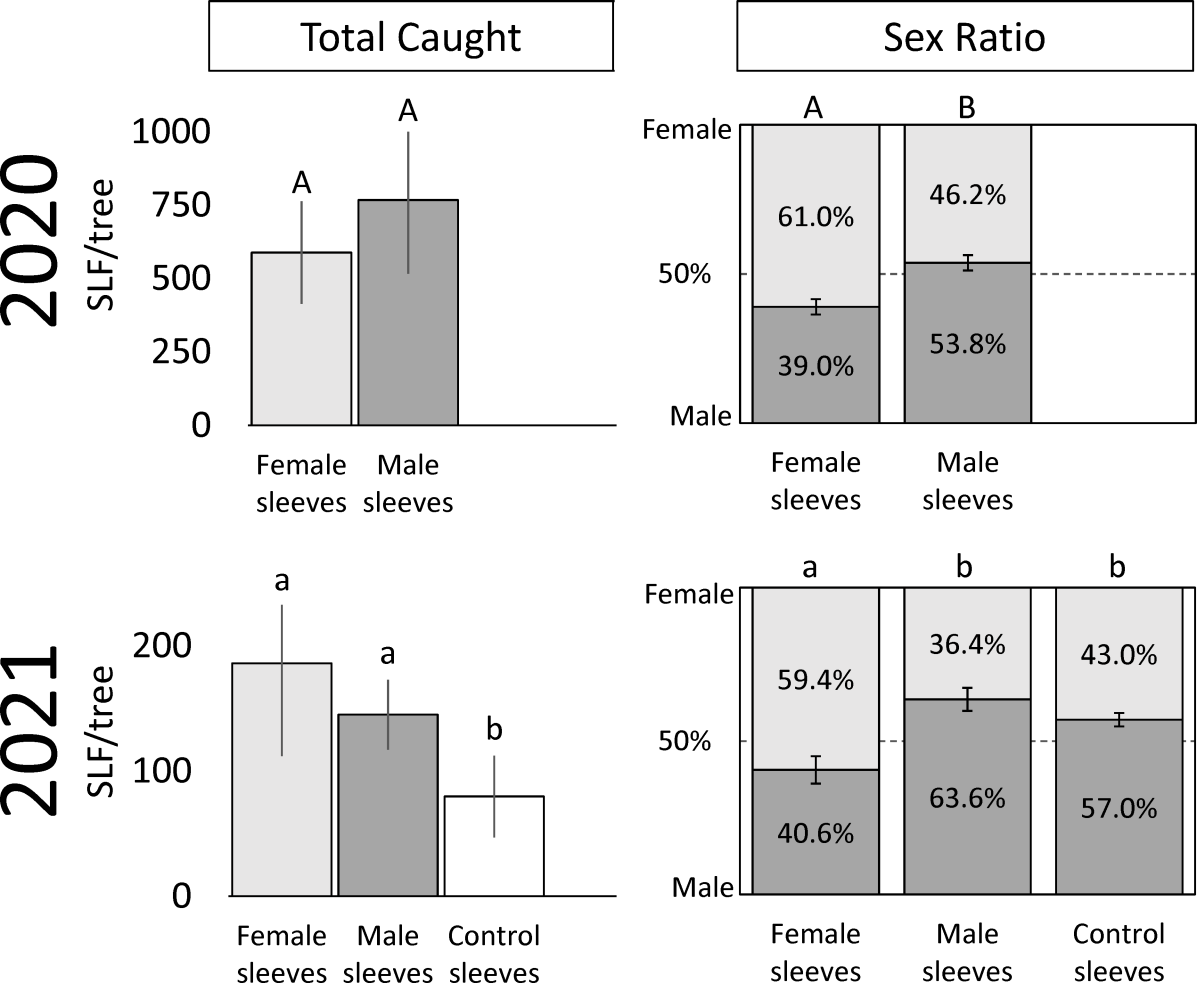
The average numbers ( ± SE) of total free-living adult spotted lanternflies, *Lycorma delicatula* (SLF) captured, and their overall sex ratios, on trees outfitted with sleeves containing artificial aggregations of either SLF males or females, or containing no SLF (control sleeves) over the entire trapping period in 2020 and 2021. Within each measured variable (total SLF caught and sex ratio) and year, bars with the same letters do not differ significantly.

**Figure 3:**
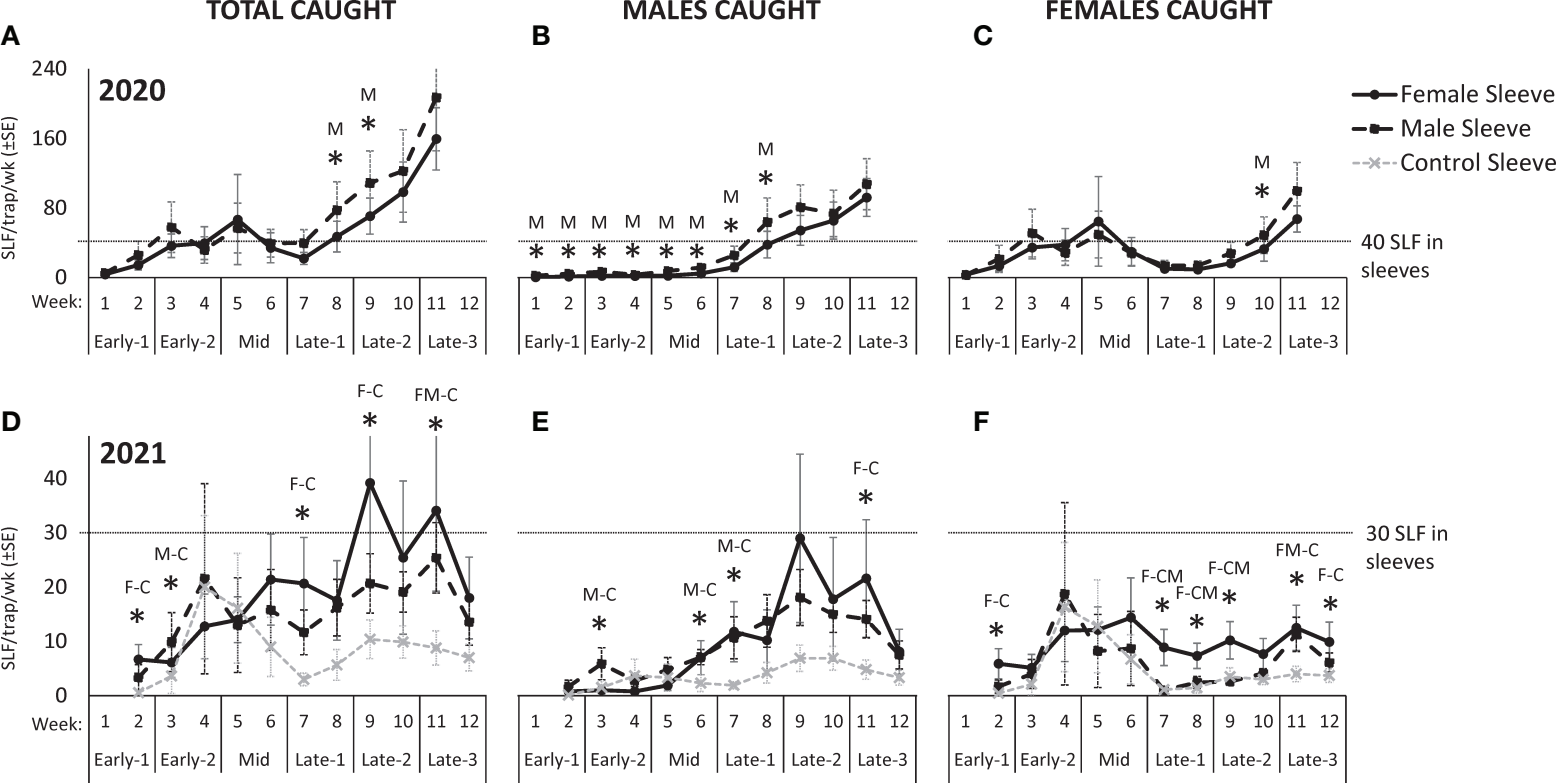
The weekly average numbers ( ± SE) of total, male, and female, respectively in 2020 **(A–C)** and 2021 **(D–F)**, of free-living adult spotted lanternflies, *Lycorma delicatula* (SLF) captured on trees outfitted with sleeves containing artificial aggregations of SLF males (M) (black dashed squares) or females (F) (black solid circles) or control sleeves containing no SLF (C) (gray dashed x). Asterisks indicate significant differences between treatments. In 2020, the letter represents which sleeves caught more. In 2021, letters of sleeves that were significantly different are separated by a dash.

In 2020, 13,567 free-living SLF were captured. Over the course of 2020, there were no significant differences in total SLF, males, or females captured on trees with sleeved males as with sleeved females, but total sex ratios differed significantly, as detailed below (**Figure 2**). Seasonal changes in trap capture of free-living total, male, and female SLF in 2020 can be seen in **Figure 3** (A, B, and C, respectively), for each treatment. At the beginning of the season, numbers of naturally occurring SLF captured per trap per week started out lower than the numbers within the sleeves, but increases of males (starting week 6) (**Figure 3B**) and females (starting week 3, and again in week 9) (**Figure 3C**) caused a surge in total SLF captured per week, exceeding the numbers in the sleeves in 2020 (**Figure 3A**). Over time, although males were captured significantly more on trees with male sleeves during weeks 1-8 (**Figure 3B**), there was no clear indication of which sex found the other sex for mating in 2020.

In 2021, which included control trees and sites with lower background densities than the prior year, 4,519 free-living SLF were captured. In 2021, significantly more males were captured on trees with male sleeves (86.6 ± 16.4) than on control trees (33.3 ± 14.2), and the number of males caught on trees with female sleeves (95.8 ± 47.2) did not differ from the other two treatments (*P* = 0.016, 0.128; F-ratio = 5.10, 1.79; df = 2, 10). Significantly more females were captured on trees with female sleeves (90.5 ± 29.1) than control trees (46.5 ± 22.9), and the number of females caught on trees with male sleeves (58.6 ± 23.9) did not differ from the other two treatments (*P* = 0.012, 0.012; F-ratio = 5.56, 3.25; df = 2, 10). In total, significantly more SLF were captured on trees with male or female sleeves than on control sleeves (*P* = 0.010, 0.083; F-ratio = 5.80, 2.05; df = 2, 10) (**Figure 2**). Thus, the artificial aggregations drew significantly more SLF to those trees than controls, and a pattern of males locating male sleeves, and females locating female sleeves, was seen.

Seasonal changes in trap capture of free-living total, male, and female SLF in 2021 can be seen in **Figure 3** (D, E, and F, respectively), for each treatment. As seen in 2020 (**Figures 3B, C**), in 2021 there was a sharp influx of males (starting week 6) (**Figure 3E**) and females (starting in week 4) (**Figure 3F**). The influx of females diminished in week 7 on male and control sleeves, but was sustained on female sleeves thereafter (**Figure 3F**). The influx of males occurred on both male and female sleeves, but not on control sleeves, and was sustained until week 12 (**Figure 3E**). In both years, the influx of females occurred in Early-2, followed by the influx of males during Mid, when mating started.

By looking at the numbers captured over time in 2021, it was again not obvious which sex attracted the other for mating, because the significant values indicated that males were more attracted to male sleeves, and females were more attracted to female sleeves. However, a difference in the behavior between males (**Figure 3E**) and females (**Figure 3F**) appears as a trend over time starting in week 6 in 2021 (Mid), in that both male and female sleeves attracted more males than control sleeves (**Figure 3E**), but only female sleeves attracted females, not male sleeves or control sleeves (**Figure 3F**). This trend suggests that females aggregated on all three treatments prior to mating and with females after mating, but males started locating aggregations (not control trees) around the time that mating started (week 6).

### Sex ratios of naturally occurring SLF

**Figure 4:**
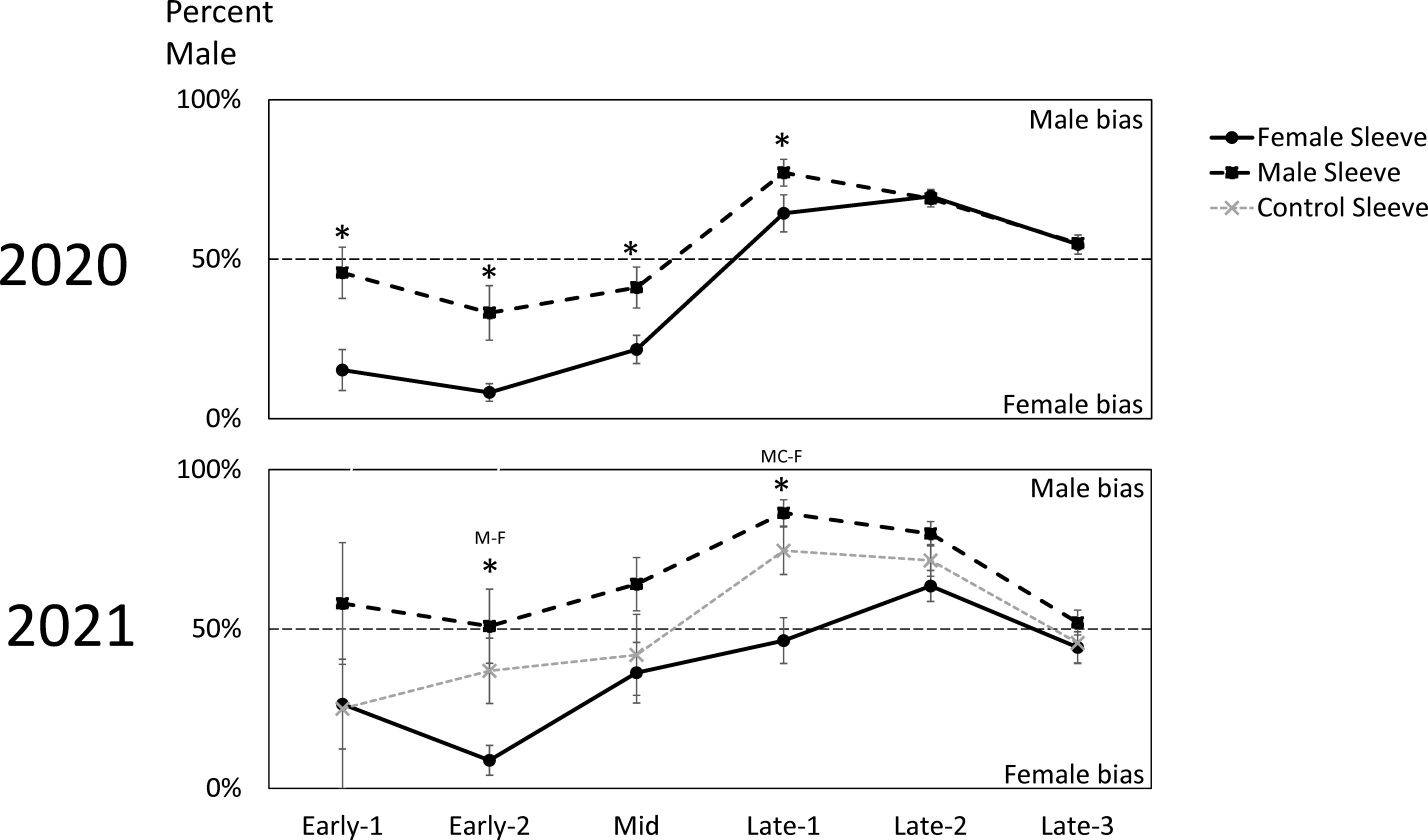
The biweekly average sex ratios ( ± SE) of naturally occurring adult spotted lanternflies, *Lycorma delicatula* (SLF) captured on trees outfitted with sleeves containing artificial aggregations of SLF males (M) (black dashed squares) or females (F) (black solid circles) or control sleeves containing no SLF (C) (gray dashed x) in 2020 and 2021. Asterisks indicate significant differences between treatments. In 2021, letters of sleeves that were significantly different are separated by a dash.

In 2020, trees with sleeved males had significantly higher sex ratios of captured SLF (53.8% male) than trees with sleeved females (39.0% male) (Paired t-test; *P* = 0.0187, t-ratio = 2.86, df = 9) (**Figure 2**). Similarly, the time sequence and total season sex ratio data suggested that each sex was most attracted to its own sex in 2020 (**Figures 2**, **4**). A similar pattern was found in 2021, where trees with sleeved males had significantly higher sex ratios (63.6% male) than trees with sleeved females (40.6% male), and control sleeves (57.0% male) which differed from female-, but not male-, sleeved trees (*P* = 0.004, 0.001; F-ratio = 7.44, 4.88; df = 2, 10) (**Figure 2**). In both years during Early-2, the sex ratio on female sleeves was less than 10% male. Even though female-sleeved trees were more female biased than male-sleeved trees, in both years the sex ratios of each treatment shifted over time in a similar pattern, from more female- to more male-biased, then converging to approximately 50% at the end of the season (**Figure 4**).

### Photographic data on SLF clusters

**Figure 5:**
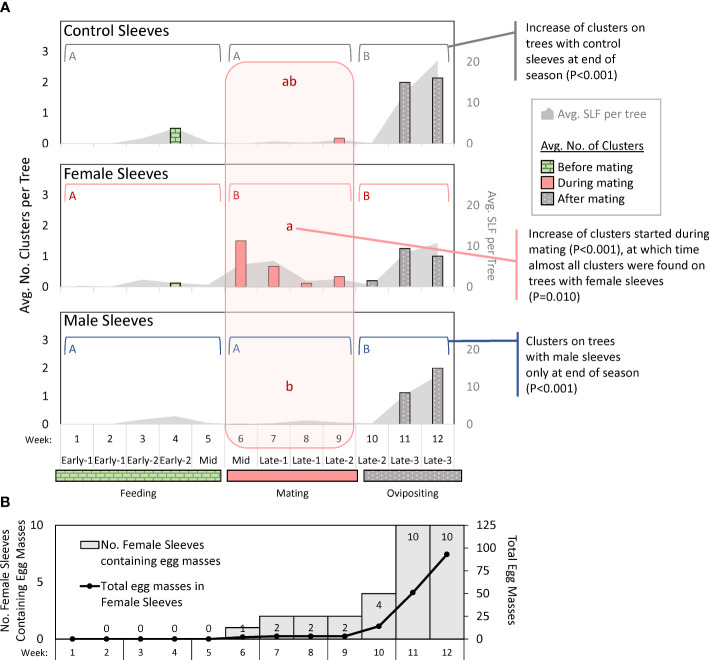
Graphs in **(A)** show weekly average numbers of clusters (columns) of adult spotted lanternflies, *Lycorma delicatula* (SLF) photographed on trees outfitted with control sleeves containing no SLF or sleeves containing artificial aggregations of 30 SLF females or males in 2021. Brackets with different uppercase letters signify differences between time intervals during which feeding, mating, or oviposition was the primary activity. The lowercase letters in the shaded vertical area signify differences between sleeve treatments during the mating period. No other period had significant differences between sleeves. The gray shaded areas represent the average numbers of free-living SLF per tree in photographs (secondary y-axis). The bottom graph **(B)** shows the number of female sleeves with egg masses (shaded), and the total number of egg masses in those sleeves (line) over time in 2021.

The numbers of free-living SLF and clusters of SLF on each tree photograph were compared for differences between sleeve treatments in each time period, and between time periods in each sleeve treatment (α = 0.025). The number of clusters changed over time for all treatments (**Figure 5A**): control sleeves (*P* < 0.001, chi-square = 18.441, df = 2), female sleeves (*P* < 0.001, chi-square = 15.69, df = 2), and male sleeves (*P* < 0.001, chi-square = 31.29, df = 2). Pairwise comparisons for each significant factor showed that for control sleeves, there were significantly more clusters during oviposition time than feeding time (*P* < 0.001, Z = -3.86, df = 2) and mating time (*P* = 0.012, Z = -2.49, df = 2); for female sleeves, there were significantly fewer clusters during feeding time than mating time (*P* = 0.001, Z = 3.24, df = 2) or oviposition time (*P* < 0.001, Z = -3.96, df = 2); and for male sleeves there were significantly more clusters during oviposition time than either feeding time (*P* < 0.001, Z = -4.68, df = 2) or mating time (*P* = 0.001, Z = -3.13, df = 2) (**Figure 5A**). During feeding and oviposition time, there were no differences between sleeve treatments, but during mating time there were significantly more clusters on female sleeves than on male sleeves (*P* = 0.010, Z = -2.59, df = 2).

The same analysis was conducted on the total number of SLF per tree in photographs, and there were no differences between sleeve treatments for any time period (α = 0.025). It was, therefore, removed from the model. Time period differed significantly in the overall model (*P* < 0.001, chi-square = 62.44, df = 2), and the number of SLF was different between feeding (score mean = 98.6) and mating (score mean = 126.4) (*P* < 0.001, Z = 3.69, df = 2), feeding and oviposition (score mean = 174.7) (*P* < 0.001, Z = -7.77, df = 2), and mating and oviposition (*P* < 0.001, Z = -4.02, df = 2).

### Egg masses inside sleeves

In 2021, no egg masses were deposited inside any control sleeves, and a free-living female SLF entered through a hole in one male sleeve and deposited a single egg mass in that sleeve in week 11, which was still present in week 12. Inside female sleeves, there were no egg masses during weeks 1 through 5. The total (and average) number of egg masses accumulating inside all female sleeves from week 6 to 12, respectively, were 2 (0.2), 3 (0.3), 3 (0.3), 3 (0.3), 14 (1.4), 51 (5.1), and 93 (9.3). In weeks 6-9, only two female sleeves contained egg masses. In week 10, only four sleeves contained egg masses. In weeks 11 and 12, all female sleeves contained egg masses (**Figure 5B**).

### Mark-release-recapture of SLF adults

**Figure 6:**
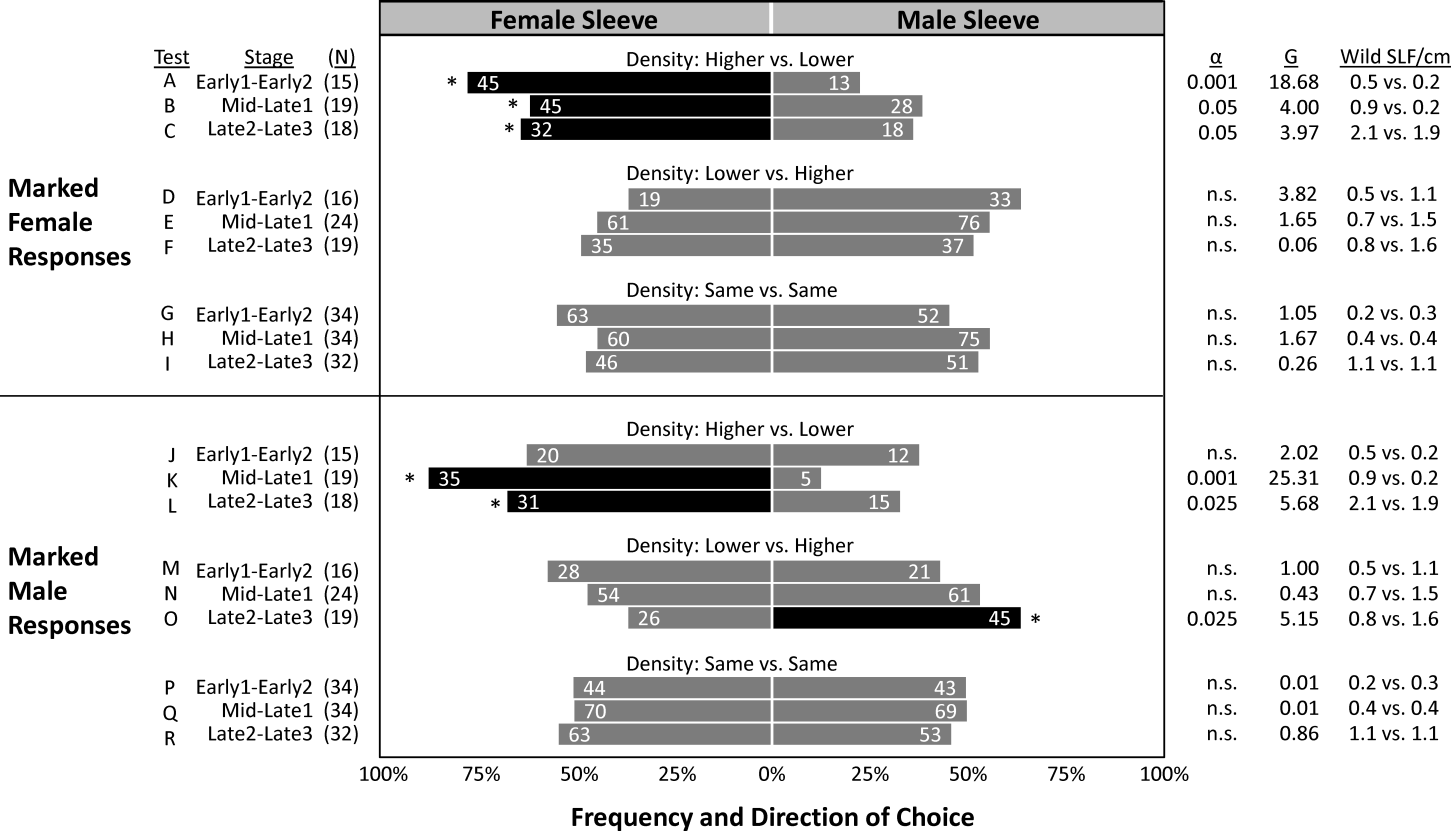
The frequency of choices made by marked-released-recaptured adult spotted lanternflies, *Lycorma delicatula* (SLF) (x-axis) of different ages in the field. In 2020 and 2021, groups of marked female and male adult SLF were released weekly halfway between pairs of trees outfitted with sleeves containing artificial aggregations of either SLF females (left) or males (right). Pairs of trees were categorized *post hoc* based on their relative naturally occurring unmarked SLF population density (wild SLF/cm circumference of the tree caught per week) relative to that on the other tree in each pair. The numbers in each bar indicate the total number of marked-released SLF that were recaptured over each 4-week trapping period. Asterisks and black shading indicate the choices that deviated significantly from predicted (Chi square test). The numbers of releases in each category are shown as (N). Critical alpha levels of significance, test statistic G, and average naturally occurring densities (number of wild SLF per cm circumference) on the female-sleeved vs. male-sleeved trees being tested are shown in columns on the right. Alpha greater than 0.05 indicates no significant difference (n.s.).

In the two years combined, a total of 6,630 SLF were captured, marked, and released, and 1,514 of those were recaptured (22.8% total recapture rate). In 2020 and 2021, 24.8% and 20.6% of marked-released SLF were recaptured, respectively (**Table 2**). The vast majority of SLF were recaptured in the first week of their release, but 14% and 4.2% of marked males and females, respectively, were recaptured in the weeks that followed (**Table 2**). The proportion of trees that caught lower numbers of unmarked SLF per week than the number of SLF inside the sleeves for Early, Mid/Late1, and Late2/Late3, were 87%, 68%, and 27%, respectively for 2020, and 92%, 86%, and 85% in 2021, indicating that most of the time the naturally occurring SLF population density was low compared to the aggregations within sleeves. Timing, relative background density, and the sex of the artificial aggregation contained within the sleeve all played a role in what choices were made by marked-released-recaptured SLF (**Figure 6**). **Figure 6** compares marked SLF responses, given a choice between two trees, taking into consideration differences in the natural SLF density occurring on the two trees within each pair, and which direction the difference was with respect to the contents of the sleeves. Relative background density interacted with sleeve choice, in that the trees with the higher relative background densities were chosen significantly more. A given tree did not have the same relative background density designation each week, thus background populations of SLF and their tree preferences fluctuated, but they did have the same sleeve designation (male or female) each week. By comparing the significant choices of marked-released SLF on the higher density trees (controlling for weekly relative background density changes), significant preferences were revealed. During Early, Mid, and Late, marked females significantly preferred trees with the higher background density when associated with sleeves containing females (**Figures 6A–C**), but not sleeves containing males (**Figures 6D–F**). Early males showed no preference for either sleeve coupled with the higher density tree (**Figures 6J, M**). During Mid, marked-released males significantly preferred the higher density tree when coupled with sleeves containing females (**Figure 6K**), but not when coupled with sleeves containing males (**Figure 6N**). During Late, marked males preferred the higher density trees regardless of the sex within the sleeve (**Figures 6L**, **O**). All other combinations resulted in no preference. Neither males nor females at any time demonstrated a sleeve preference when their background densities were equal. The naturally occurring average weekly SLF background density (wild SLF per cm circumference of the tree) for the nine comparisons is displayed in **Figure 6**.

**Table 2:** The total number of marked adult spotted lanternflies, *Lycorma delicatula* (SLF) in 2020 and 2021 combined that were recaptured, the overall recapture rates of females and males during 4-week time periods, and the number of weeks after which different proportions of recaptures occurred.

	Females	Males
Total SLF recaptured	789	695
Overall recapture rates (%)		
Early1-Early2	27.3	20.4
Mid-Late1	23.7	20.2
Late2-Late3	21.2	22.5
Weeks after release (%)		
1	95.7	86.1
2	2.0	7.4
3	1.1	3.5
4+	1.1	3.1

## Discussion

Our main objectives were to determine who finds whom and when among SLF, and if we can use artificial aggregations to manipulate natural aggregations. The experiments demonstrated that, overall, adult SLF oriented significantly to confined artificial aggregations of other adult SLF. Specifically, caged aggregations of males drew significantly more free-living male SLF than controls, and caged aggregations of females drew significantly more free-living female SLF than controls. As such, in both 2020 and 2021, sex ratios (percent male) of free-living SLF were significantly more male-biased on male sleeves and female-biased on female sleeves (**Figure 2**). This evidence suggests that the natural male- and female-biased sex ratios that have been previously reported on different trees (12), likely form at least partly in response to sex-specific conspecific signals. The fact that the female sleeve treatment had significantly more female-biased sex ratios than male or control treatments, which were similar to each other, suggests that signals produced from females aggregating on *A. altissima* attracted more females. Such signals could be derived from the insects themselves, or from an interaction between the insects and their host plant. Logistical considerations precluded adding an additional treatment to test mixed-sex artificial aggregations, which is also of interest. However, due to the abrupt shift we have repeatedly observed in naturally occurring sex ratios from relatively unbiased during Early-1, to extremely male- or female-biased on different trees in the same vicinity which we use to characterize the Early-2 phase, it was decided that measuring SLF responses to same-sex aggregations was the primary question for this study.

Looking at the capture data over time, some interesting trends and differences are revealed (**Figure 3**). In both years, free-living females started arriving and becoming captured in large numbers on all treatments during Early, approximately two weeks prior to mating (**Figures 3C**, **F**). Captures of arriving males started to surge two weeks later during Mid, when mating began, and only on trees with artificial aggregations (**Figures 3B**, **E**). This difference in arrival time between naturally occurring females and males is reflected in the sex ratio shifts over time seen in both years (**Figure 4**), where sex ratios were more female-biased during Early. Around mating time (Mid), sex ratios approached 50% (**Figure 4**), and arriving females started showing a significant preference for confined females over confined males or controls (**Figure 3F**). After oviposition was observed (Late), female arrivals somewhat diminished (**Figures 3C**, **F**) and male arrivals continued to increase (**Figures 3B**, **E**), resulting in a male bias during Late (**Figure 4**).

In the mark-release-recapture experiment, we cannot remove the influence that the naturally occurring population may have had on marked SLF, nor can we separate the effects of the sleeves on that naturally occurring population. However, we can analyze tree pairs based on the combination of those factors and look at their combined effects on the choices of marked SLF. In doing so, attraction to the tree in the pair with the higher density natural SLF population was observed as interacting with the sleeve treatments, in which marked-released adult SLF distinguished between sleeves containing either males or females only when that sleeve coincided with the tree with the higher background density. Corresponding with the timing of the natural surge in female arrivals, marked-released Early females significantly and most strongly preferred higher density trees only when combined with female aggregations (**Figure 6A**) but not with male aggregations (**Figure 6D**). This significant attraction of marked females to higher density trees with confined females, but not confined males, continued into Mid and Late, but was most pronounced during Early. No significant preferences were found in marked Early males. Corresponding with the timing of the later surge in males during mating time, marked-released Mid males significantly and most strongly preferred higher density trees when combined with confined females (**Figure 6K**) but not with confined males (**Figure 6N**), showing a strong attraction of marked Mid males to Mid females. Males during the Late stages, when natural populations were higher and sleeved females were unlikely to have been sexually receptive (as indicated by oviposition inside sleeves), significantly chose the tree with the higher background population density regardless of the sleeve contents (**Figures 6L**, **O**). When the higher background population density was on the male trees, there was little effect of the sleeves on choices of marked SLF. Curiously, in the absence of background population density differences between trees (**Figures 6**, **G–I**, **P–R**), sleeve contents had no effect on choices of marked SLF, leaving some unanswered questions as to why sleeves containing males or females were able to influence the naturally occurring population, but not marked individuals released midway between paired trees. Thus, the results do not explain all of the observed behaviors and additional work is still needed to fully decipher how SLF make decisions when locating each other for mating or aggregation. From the significant trapping results of naturally occurring SLF and marked-released SLF captured over time, it appears that females locate females for aggregation and feeding, and males locate female aggregations for mating. Aggregation in insects is not defined by a single set of behaviors or mechanisms, and although attraction can play a role in aggregation, at the other end of the spectrum aggregation can result from random movements combined with arrestment (23, 24). Thus, a variety of different behavioral mechanisms may result in aggregation. Although these field experiments describing SLF aggregations over time in response to artificial same-sex aggregations provide key information about who finds whom and when, and demonstrate that aggregations can be manipulated, more work is needed to determine how aggregations are initiated or the mechanisms used to aggregate.

In 2020, the free-living SLF population density became much higher than the numbers of SLF in the sleeves, likely influencing the results that year (**Figure 3A**). However, the lower population densities in 2021 allow a look at SLF responses with less influence from naturally occurring populations (**Figure 3D**). In 2021 over time, especially after week 5 (Mid), more males were caught on male sleeves than controls (**Figure 3E**), and more females were caught on female sleeves than control or male sleeves (**Figure 3F**). The presence of the control trees in 2021 revealed a trend that, once mating had begun, males consistently oriented to both male and female sleeves more than controls (**Figure 3E**), but females oriented to female sleeves, not male sleeves or controls (**Figure 3F**). Thus, attraction was not symmetrical between sexes in that males were attracted to both males and females but females were attracted to only females. This likely resulted in the observed male- and female-biased populations of SLF on different trees. Such SLF sex ratio biases in the weeks leading up to mating have previously been described in natural populations (12, 13). The asymmetry in attraction speaks to the complexity of this system, suggesting multiple signalers and receivers, with potentially multiple sensory modalities involved, and illustrates how SLF attraction and aggregation behavior will not be fully conveyed by simple explanations.

Our field data on long range attraction corroborates results from laboratory walking olfactometer bioassays testing attraction to SLF-derived volatiles, giving evidence to suggest these behaviors may be mediated by pheromones to some degree (10, 16). Olfactometer studies found that male SLF were attracted to volatiles only from male-produced honeydew, and although not significant, females trended towards attraction to honeydew from females, but not males (10). In olfactometer studies on SLF body volatile extracts, we found that Early males were attracted to body volatiles from both sexes, but females were not (16). In that study, Mid males were able to distinguish between the body volatiles of Mid males and females and were attracted only to the volatile extracts from females. Therefore, a proposed set of mechanisms for the observed field attraction of males and females at different times is starting to materialize in which both body volatiles and honeydew volatiles from male and female SLF may play sex-specific roles in attraction for the purposes of aggregation and mating. This does not exclude the possible use by SLF of other conspecific communication mechanisms or signals, such as the release of plant damage volatiles from feeding activity, or substrate vibrations, which are commonly used by other members of Hemiptera to form aggregations or locate mates (25). However, substrate vibrations are limited spatially in that the signaler and receiver typically must already be on the same substrate, and signals attenuate beyond a few meters (26–28).

The trapping studies did not evaluate arrestment or aggregation behavior because they measured differences in the numbers of SLF that arrived on tree trunks, which is a measure of attraction. What happened after SLF arrived, such as arrestment or courtship, could be captured by the photographic data, which provided snapshots of their positions and behavior over time. Photographs informed us of where and when clustering, our measure of courtship, took place. This was defined as groups of two or more SLF that were physically in contact, often positioned in parallel or in groups, with bodies touching. Clustering during mating time was almost exclusively on trees with female sleeves (**Figure 5A**). Superficially, this side-by-side pairing of male and female SLF during mating time (see 9, 12) appears similar to whitefly courtship behavior in which a combination of a short range sex pheromone and substrate vibrations are employed (29–31). In the final two weeks of the study, when egg masses had been deposited in all female sleeves (**Figure 5B**), the naturally occurring population of SLF increased on all sleeve treatments, as did clustering (**Figure 5A**). During this time, the increased numbers of free-living SLF on trees may have exceeded any effects of the sleeved SLF. It is unclear what drove this increase in SLF and clustering when oviposition was well underway. It is possible that females, having fed and mated, left depleted trees seeking oviposition sites, and that aggregation continues to occur throughout this process. If so, it could explain why egg masses can also be observed in clusters (KM, pers. obs.). Although snapshots of clustering behavior and SLF on trees showed an increase in all sleeve treatments by week 11, this was not reflected in weekly trapping data which indicated that female sleeves still captured the most SLF, followed by male sleeves, and then controls at that time (**Figures 3D**–**F**). What guides SLF behaviors during their oviposition period should be investigated further, but it was not the focus of this study.

The scarcity of data currently available on fulgorid chemical ecology can be attributed to a lack of exploration. Until the recent invasions of SLF in Korea (2004), Japan (2008), and the United States (2014) (32), fulgorid chemical ecology had been neglected in the literature. There are numerous examples in the literature of pheromone use within the three major suborders of Hemiptera. Most examples are in Heteroptera (true bugs) (see reviews by 33, 34), and some are known from Sternorrhynca which includes aphids (35), whiteflies (29), scales (36), mealybugs (37), and psyllids (38). Pheromone use has even been documented in the suborder to which SLF belongs, Auchenorrhyncha, which contains cicadas, treehoppers, leafhoppers, planthoppers, and spittle bugs (39), although it is widely understood that this suborder relies heavily on sound or substrate vibrations to locate mates (25). More research describing the sensory ecology of SLF is critical to the success of any control program.

## Data availability statement

The raw data supporting the conclusions of this article will be made available by the authors, without undue reservation.

## Author contributions

MC secured funding, conceived and designed the experiments, oversaw the experiments, analyzed the data, and wrote the manuscript. KM conducted the field experiments, oversaw and coordinated the field work, handled field logistics, and collected the data. All authors contributed to the article and approved the submitted version.

## Funding

We thank the Plant Protection Act 7721 for funding projects 3.0106 and 3.0792 in 2020 and 2021, respectively, which helped support this work and the USDA and cooperator personnel who contributed (AP20PPQS&T00C030, AP20PPQS&T00C023, AP21PPQS&T00C117, AP21PPQS&T00C119).

## Acknowledgments

This work could not have been conducted without the care, dedication, and help of numerous people. We are extremely grateful for the technical support provided by Stefani Cannon, Sebastian Harris, Kyle Kaye, Levi Morris, Aubrianna Stetina, Reannon Zangakis, Kerry Handelong, Cole Davis, Jeremy Rapposelli, Isaiah Canlas, Sam Stella, Matthew Wallace, Annie Ray, East Stroudsburg University, and Xavier University. Our sincere gratitude goes to the many property owners who provided access to their land for these studies. Our gratitude goes to Melissa Warden for her time and discussions on the statistical analysis, and to Hajar Faal, Allard Cossé, and Joe Francese for their thoughtful discussions and insights, and the anonymous reviewers who provided valuable critique. This material was made possible, in part, by a Cooperative Agreement from the United States Department of Agriculture’s Animal and Plant Health Inspection Service (APHIS). It may not necessarily express APHIS’ views.

## Conflict of interest

The authors declare that the research was conducted in the absence of any commercial or financial relationships that could be construed as a potential conflict of interest.

## Publisher’s note

All claims expressed in this article are solely those of the authors and do not necessarily represent those of their affiliated organizations, or those of the publisher, the editors and the reviewers. Any product that may be evaluated in this article, or claim that may be made by its manufacturer, is not guaranteed or endorsed by the publisher.
